# Vancomycin bone and tissue concentrations following tibial intraosseous administration – evaluated in a porcine model

**DOI:** 10.5194/jbji-6-99-2021

**Published:** 2021-02-12

**Authors:** Josephine Olsen Kipp, Pelle Hanberg, Josefine Slater, Line Møller Nielsen, Stig Storgaard Jakobsen, Maiken Stilling, Mats Bue

**Affiliations:** 1 Orthopaedic Research Unit, Aarhus University Hospital, Palle Juul-Jensens Blvd. 99, 8200 Aarhus N, Denmark; 2 Department of Orthopaedic Surgery, Horsens Regional Hospital, Sundvej 30, 8700 Horsens, Denmark; 3 Department of Clinical Biochemistry, Aarhus University Hospital, Palle Juul-Jensens Blvd. 99, 8200 Aarhus N, Denmark; 4 Department of Orthopaedic Surgery, Aarhus University Hospital, Palle Juul-Jensens Blvd. 99, 8200 Aarhus N, Denmark

## Abstract

**Introduction**. Systemic perioperative vancomycin may not provide sufficient prophylactic target-site concentrations in the prevention of prosthetic joint infections. Intraosseous vancomycin potentially provides high target-site concentrations. The objective of the present study was to evaluate the local bone and tissue concentrations following tibial intraosseous vancomycin administration in a porcine model. **Methods**. Eight pigs received 500 mg diluted vancomycin (50 mg/mL) through an intraosseous cannula into the proximal tibial cancellous bone. No tourniquet was applied. Microdialysis was applied for sampling of vancomycin concentrations in adjacent tibial cancellous bone, in cortical bone, in the intramedullary canal of the diaphysis, in the synovial fluid of the knee joint, and in the subcutaneous tissue. Plasma samples were obtained as a systemic reference. Samples were collected for 12 h. **Results**. High vancomycin concentrations were found in the tibial cancellous bone with a mean peak drug concentration of 1236 (range 28–5295) µg/mL, which remained high throughout the sampling period. The mean (standard deviation) peak drug concentration in plasma was 19 (2) µg/mL, which was obtained immediately after administration. Peak drug concentration, time to peak drug concentration, and area under the concentration–time curve were within the same range in the intramedullary canal, the synovial fluid of the knee, and the subcutaneous tissue. **Conclusion**. Tibial intraosseous administration of vancomycin provided high concentrations in tibial cancellous bone throughout a 12 h period but with an unpredictable and wide range of peak concentration. The systemic absorption was high and immediate, thus mirroring an intravenous administration. Low mean concentrations were found in all the remaining compartments.

12 February 2021

## 1 Introduction

Prosthetic joint infection (PJI) is a serious and costly complication to joint replacement surgery (Hackett et al., 2015). The economic cost of a PJI is of significant influence for both the patient and the healthcare system, associated with increased morbidity, prolonged hospital admission length, and decreased functional recovery (Hackett et al., 2015; Zmistowski et al., 2013; Lenguerrand et al., 2017). Perioperative antibiotic prophylaxis is an obligate tool in the prevention of PJI. A successful perioperative antibiotic prophylaxis is dependent on achievement of therapeutic antibiotic target-site concentrations throughout the surgical procedure (Bryson et al., 2016; Mangram et al., 1999). In patients with high risk of developing methicillin-resistant *Staphylococcus aureus* (MRSA) infections, perioperative vancomycin prophylaxis is recommended (Bratzler and Houck, 2004). However, studies have shown that systemic vancomycin administration may not provide sufficient bone and tissue concentrations in some orthopaedic settings (Bue et al., 2015, 2017; Bryson et al., 2016; Mangram et al., 1999). Local vancomycin application has the potential to bypass these concerns by delivering high target-site concentrations without reaching toxic side-effects (Sweet et al., 2018; Whiteside, 2016; Young et al., 2014; Wahl et al., 2017). Several local vancomycin applications and carriers are currently available, e.g. powder, calcium sulfate, and irrigation. Particularly in implant surgery, local vancomycin application may be promising to prevent bacterial implant colonization (Whiteside, 2016; Young et al., 2014).

Local vancomycin administration as an addition to systemic antibiotics has been shown to reduce the infection rates in several orthopaedic settings such as tibial fractures and spine surgeries (Craig et al., 2014; Xie et al., 2017). Intraosseous vancomycin administration into the proximal tibia is an easy and feasible local vancomycin application. Intraosseous vancomycin has previously been employed as a perioperative antibiotic prophylaxis in total knee replacement and revision surgery and suggested as enhancing the effectiveness of the perioperative antibiotic prophylaxis (Young et al., 2014; Harper et al., 2020; Young et al., 2018; Chin et al., 2018). These studies assessed vancomycin tissue concentrations by means of tissue specimens, which, however, is limited by its invasiveness, static sampling points, determination of the total, and not free drug concentration. In addition, the concentrations in tissue samples are measured in a homogenate that may include lingered blood and do not appreciate the different tissue compartments. Microdialysis is an alternative method to tissue specimens and is significantly advantaged by allowing for continuous sampling of the active concentrations of vancomycin simultaneously from multiple orthopaedic-relevant sites (Joukhadar and Muller, 2005; Muller, 2002). The objective of this study was to evaluate the local tissue concentrations over a 12 h period after tibial intraosseous vancomycin administration using microdialysis in a porcine model.

## 2 Methods

The study was conducted at the Institute for Clinical Medicine, Aarhus University Hospital, Aarhus, Denmark. All chemical analyses were performed at the Department of Clinical Biochemistry, Aarhus University Hospital, Aarhus, Denmark. The study involved animals and all the procedures were in accordance with the ethical standards of the institution (the Danish Animal Experiments Inspectorate, license no. 2017/15-0201-01184).

### 2.1 Animals, anesthesia, and surgical procedures

Eight female pigs, from the same supplier and raised under the same conditions, were included in the study (Danish Landrace Breed, age 5 months, weight 78–82 kg). The pigs were kept under general anesthesia with a continuous infusion of propofol (450–650 mg/h) and fentanyl (0.5–0.7 mg/h) throughout the entire study period. The arterial pH and body temperature were monitored and kept in the ranges of 7.37–7.52 and 36.4–40.0 ${}^{\circ }$C, respectively. After the study, the pigs were euthanized using pentobarbital.

**Figure 1:**
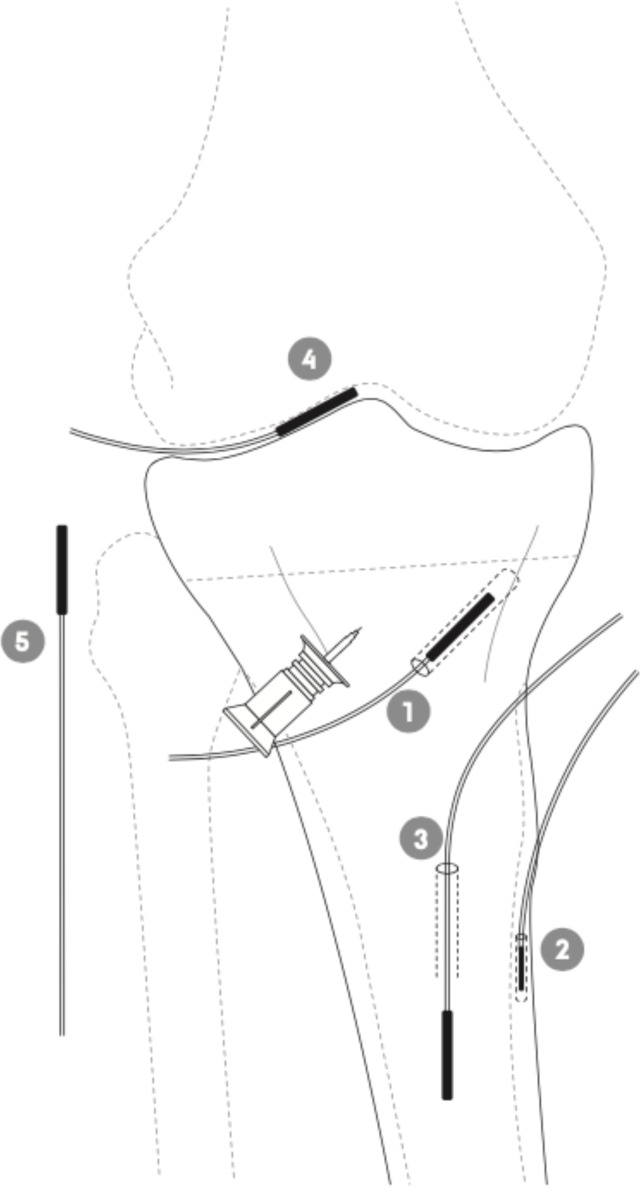
Schematic illustration of the placement of the tibial intraosseous cannula and the microdialysis probes in adjacent cancellous bone (1), cortical bone (2), intramedullary canal (3), synovial fluid of the knee joint (4), and subcutaneous tissue (5) in a porcine model.

An intraosseous cannula (15 mm) was inserted into the medial tibial condyle approximately 10 mm distal to the epiphyseal line; see Fig. 1. Three tibial microdialysis probes were placed in drill holes, one in the adjacent cancellous bone parallel to and at a mean distance of 9 (range 7–10) mm from the intraosseous cannula tip (drill depth 35±1 mm), one in the cortical bone at the anterior–medial margin of the tibial diaphysis (drill depth 14±0.5 mm), and one within the intramedullary canal of the tibial diaphysis. All drill holes were made with a 2 mm drill, which was paused every few seconds to prevent overheating of the bone tissue. Subsequently, a probe was placed in the synovial fluid of the knee joint using a splittable introducer. Finally, a subcutaneous tissue probe was placed lateral to the knee joint. No tourniquet was applied. Correct positions of all the probes and the distance between the intraosseous cannula and the adjacent cancellous bone microdialysis catheter were verified by fluoroscopy. Placement and length of all drill holes were visualized for control by a postmortem computed tomography (CT) scan, which confirmed that all drill holes were placed as intended.

### 2.2 Microdialysis

Microdialysis is a membrane-bearing probe technique allowing for dynamic sampling of molecules from the interstitial space from the tissue of interest (Joukhadar and Muller, 2005; Muller, 2002; Tottrup et al., 2019; Hanberg et al., 2019). Due to continuous perfusion of the microdialysis system, a complete equilibrium across the semipermeable membrane will never occur. The concentration in the dialysate will therefore only represent a fraction of the absolute tissue concentration, which is expressed as the relative recovery. Thus, relative recovery must be determined to assess the absolute tissue concentrations. In the present study, all microdialysis probes were individually calibrated in the beginning of the study using the retrodialysis by drug method (Scheller and Kolb, 1991).

In the present study we used CMA 107 precision pumps (μ-Dialysis AB, Stockholm, Sweden) producing a flow rate of 1 µL/min and CMA 70 probes (membrane lengths 10 and 20 mm, molecular cutoff 20 kDa).

### 2.3 Sampling procedures

Immediately after placement, the probes were perfused with isotonic saline containing 2.5 µg/mL vancomycin, followed by a 30 min tissue equilibration period. Afterwards, all probes were individually calibrated by the retrodialysis method by collecting a 60 min sample. The perfusate was then changed to isotonic saline followed by a 180 min washout period in order to prevent calibration leftovers of vancomycin. A vancomycin (500 mg) and saline (10 mL) dilution at 50 mg/mL was injected over 30 s at time zero through the intraosseous cannula and flushed with 3 mL saline. The first three dialysates were collected at times 40, 80, and 120 min. Thereafter, dialysates were collected at 60 min intervals from times 120 to 720 min, giving a total of 13 samples from each compartment over 12 h. Blood samples were drawn from a central venous catheter at the midpoint of the microdialysis sample intervals. The dialysates were instantly stored at -80 ${}^{\circ }$C until analysis. Blood samples were stored at 5 ${}^{\circ }$C for a maximum of 4 h before being centrifuged at 3000 rpm for 10 min. The obtained plasma aliquots were then stored at -80 ${}^{\circ }$C until analysis.

### 2.4 Vancomycin quantification

The free concentration of vancomycin in plasma was quantified with a homogeneous enzyme immunoassay technique using the Siemens Chemistry XPT platform (Advia Chemistry, Erlangen, Germany). Intra-run (total) imprecisions for the assay were ±1.2 µg/mL (2 standard deviations – SD) at 6.6 and ±3.7 µg/mL (2 SD) at 29.1 µg/mL. The vancomycin concentrations in the dialysates were quantified using ultra-high performance liquid chromatography (Bue et al., 2015). The intra-run (total) imprecisions (percent coefficient of variation – % CV) were evaluated at three different vancomycin concentrations: 3.7 % (5.7 %) at 0.7 g/mL, 3.0 % (3.5 %) at 3.7 g/mL, and 0.9 % (2.2 %) at 5.2 g/mL. The quantification limit was defined as the lowest concentration with intra-run CV <20 % and was found to be 0.05 µg/mL.

### 2.5 Statistics

Standard pharmacokinetic parameters were determined separately for each compartment in each pig by non-compartmental analysis using Stata (v. 14.1, StataCorp LLC, College Station, TX, USA). The area under the concentration–time curve (AUC${}_{0-\mathrm{last}}$) was calculated using the trapezoidal rule. The maxima of all the recorded concentrations were defined as peak drug concentration ($C_{\max}$) and enabled a calculation of the time to $C_{\max}$ ($T_{\max}$). Half-life ($T_{1/2}$) was determined for all compartments except for tibial cortical bone and calculated as ln(2)/$\lambda_{\mathrm{eq}}$, where $\lambda_{\mathrm{eq}}$ is the terminal elimination rate constant estimated by linear regression of the log concentration on time. For AUC${}_{0-\mathrm{last}}$ and $C_{\max}$, a mixed model for repeated measurements was applied, taking the variance between pigs into account. The model assumptions were tested by visual diagnosis of residuals, fitted values, and estimates of random effects. A correction for degrees of freedom due to small sample size was handled using the Kenward–Roger approximation method. The t test was used to determine relevant pairwise comparisons. A p value <0.05 was considered to be significant. A Spearman test was used to test the correlation between the distance of the intraosseous cannula and the adjacent cancellous bone drill hole with the peak drug concentration. Statistical analyses were also performed using Stata. The mean values with the following SD of AUC${}_{0-\mathrm{last}}$, $C_{\max}$, $T_{\max}$, and $T_{1/2}$ are presented in Table 1. The dialysate concentrations were ascribed to the midpoint of the sampling intervals. Values below the quantification limit were set to zero. The washout concentrations were low and therefore not included in the analysis model.

**Table 1:** Key pharmacokinetic parameters given as a mean (SD) for tibial cancellous bone, tibial cortical bone, the intramedullary canal in the tibia, the synovial fluid of the knee joint, subcutaneous tissue, and plasma.

Tissue	AUC${}_{-\mathrm{last}}$ (min µg/mL)	$C_{\max}$ (µg/mL)	$T_{\max}$ (min)	$T_{1/2}$ (min)
Tibial cancellous bone	374 637 (513 172)	1236 (1883)	111 (94)	279 (188)
Tibial cortical bone	326 (331)	1 (1)	405 (278)	
Intramedullary canal in the tibia	1980 (908)${}^{a}$	8.2 (4)${}^{a}$	65 (26)	339 (332)
Synovial fluid of the knee joint	1565 (758)${}^{a}$	6.5 (4)${}^{a}$	45 (21)	217 (140)
Subcutaneous tissue	1881 (691)${}^{a}$	7.7 (3)${}^{a}$	70 (19)	361 (269)
Plasma	3,673 (611)	19 (2)	20 (19)	254 (128)

AUC${}_{0-\mathrm{last}}$: area under the concentration–time curve from 0 to the last measured value; $C_{\max}$: peak drug concentration; $T_{\max}$: time to $C_{\max}$; $T_{1/2}$: half-life at phase. ${}^{a}$ p>0.35 for all pairwise comparisons of means between the intramedullary canal, synovial fluid of the knee joint, and subcutaneous tissue.

**Figure 2:**
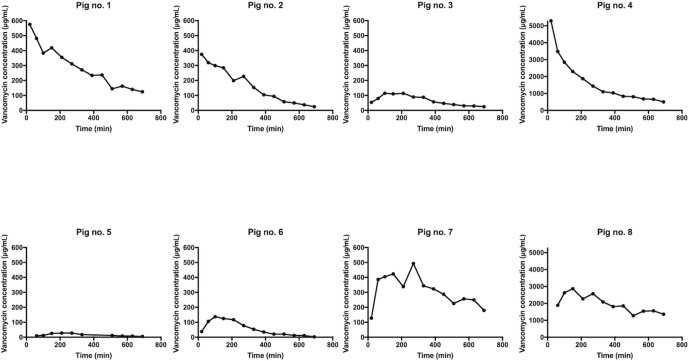
Individual concentration–time profiles for tibial cancellous bone adjacent to the intraosseous cannula in pig nos. 1–8. The y axis for pig nos. 4 and 8 is 10-fold higher than the rest.

**Figure 3:**
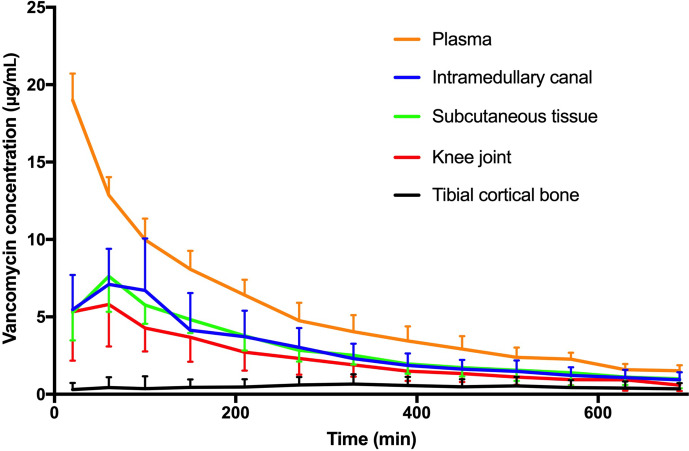
Mean concentration–time profile for plasma, intramedullary canal, subcutaneous tissue, synovial fluid of the knee joint, and tibial cortical bone. Bars represent standard deviation.

**Figure 4:**
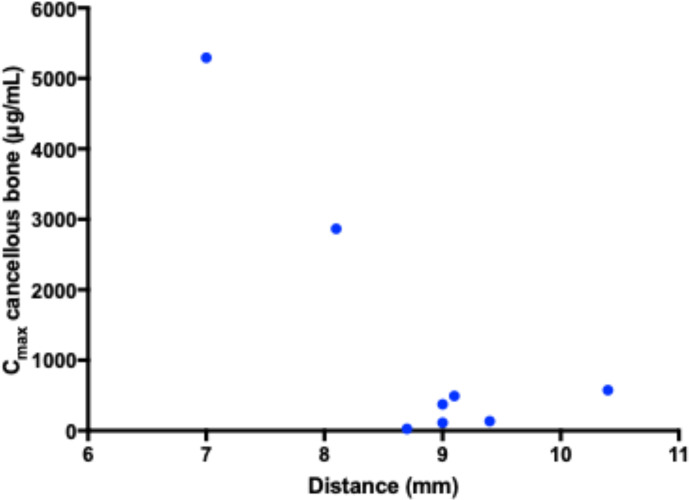
Correlation between the distance from the intraosseous cannula and the adjacent cancellous bone drill hole with the peak drug concentration. No correlation was found.

## 3 Results

All eight pigs completed the study and all the probes were functioning. Mean (SD) relative recovery was 41.5 % (10.7 %) in the tibial cancellous bone, 45.5 % (7.1 %) in the tibial cortical bone, 44.4 % (8.2 %) in the intramedullary canal, 55.3 % (19.4 %) in the synovial fluid of the knee joint, and 46.7 % (11.1 %) in subcutaneous tissue.

All the pharmacokinetic parameters are displayed in Table 1. Individual concentration–time profiles of vancomycin in the tibial cancellous bone are displayed in Fig. 2. The mean concentration–time profiles for the remaining compartments are presented in Fig. 3. The mean vancomycin $C_{\max}$ (SD) for tibial cancellous bone was 1236 (1883) µg/mL with a range of 28–5295 µg/mL. There was no statistically significant correlation between the distance from the intraosseous cannula to the adjacent cancellous bone drill hole and the $C_{\max}$ (Fig. 4). Two pigs presented approximately 10-fold higher tibial cancellous bone concentrations and one pig approximately 10-fold lower in comparison to the remaining pigs (Fig. 2). After 11.5 h (690 min), the mean (SD) vancomycin concentration was 278 (range 3–1363) µg/mL in tibial cancellous bone. For the remaining compartments, the highest $C_{\max}$ (SD) of 19 (2) µg/mL was found in plasma, which was achieved immediately after administration, while the lowest $C_{\max}$ (SD) of 1 (1) µg/mL was found in tibial cortical bone. For the intramedullary canal, the synovial fluid of the knee joint, and subcutaneous tissue, comparable AUC and $C_{\max}$ were found.

## 4 Discussion

We performed a 12 h dynamic evaluation of the local bone and tissue concentrations following intraosseous administration of 500 mg diluted vancomycin into the proximal tibial cancellous bone. The main finding was a high mean peak drug concentration of 1236 µg/mL in the adjacent tibial cancellous bone with an unpredictable and large inter-pig variation. Two pigs presented approximately 10-fold higher tibial cancellous bone concentrations and one pig approximately 10-fold lower in comparison to the rest. The end mean (SD) concentration in adjacent cancellous bone was 278 (469) µg/mL.

The large inter-pig difference in vancomycin concentrations in cancellous bone cannot readily be explained. We only sampled from one drill hole in the cancellous bone adjacent to the intraosseous cannula. The postmortem CT scan verified similar distance and no direct bone communication between the intraosseous cannulas and cancellous drill holes. Although microdialysis is a dynamic sampling technique displaying low intra-pig cancellous bone variation, the sampling site is static, which prohibits the possibility of encompassing the complete variation in the local dilution potential in the entire proximal tibial cancellous bone. Furthermore, the local dilution potential may be influenced by individual bone factors such as density and architecture. Our findings may therefore, to some extent, illustrate the challenge of controlling the local concentration of vancomycin, when vancomycin is administered locally in diluted form, rather than inadequacy of the applied method. However, this needs further investigation.

Systemic perioperative vancomycin may not provide sufficient prophylactic target-site concentrations exceeding relevant minimal inhibitory concentrations (MICs) in the prevention of PJI (Bue et al., 2015). In total knee replacement patients, sufficient vancomycin tissue concentrations were reached with a substantial delay or not at all, particularly in cortical bone when applying a standard prophylactic dosage of 1 g of vancomycin (Bue et al., 2017). Intraosseous vancomycin administration has the potential to overcome this concern by providing high target-site concentrations. In order to achieve adequate perioperative antibiotic prophylaxis, it is recommended that target-site concentration exceed the MIC values of relevant bacteria throughout the surgical procedure (Bryson et al., 2016; Mangram et al., 1999). Commonly encountered bacteria in orthopaedic infections exhibit planktonic MIC in the range of 0.5–4 µg/mL for vancomycin (Rybak, 2006; EUCAST, 2019). Using a high target limit of 4 µg/mL for all investigated extravascular compartments, sufficient mean vancomycin concentrations were only found in tibial cancellous bone for all pigs throughout the 12 h sampling interval, whereas the remaining compartments did not reach the target limit in all subjects. Our findings suggest that tibial intraosseous administration of 500 mg diluted vancomycin, without the use of a tourniquet, provides high but unpredictable tissue concentrations in the adjacent proximal tibial cancellous bone. Whether our findings are applicable to other anatomical bone locations needs further investigation.

In plasma, the mean (SD) peak vancomycin concentration of 19 (2) µg/mL was achieved immediately after administration, demonstrating a prompt absorption to the systemic circulation. These findings are consistent with the general indication for the use of tibial intraosseous administration. Interestingly, the plasma variations of vancomycin were much lower than those found in tibial cancellous bone. This may indicate a uniform systemic distribution, whereas the local dilution potential and thus maintenance of a high local vancomycin concentration in the proximity of the intraosseous cannula varied to a great extent. The local dilution potential is dependent on and determined by various factors, e.g. the surface area of the cancellous bone and the volume and composition of the encircling bone marrow. As demonstrated by other antibiotic microdialysis studies, bone tissue may present as a difficult diffusion medium for antibiotics, especially if the bone is infected or inflamed (Jensen et al., 2017; Bue et al., 2019; Thomassen et al., 2020). This may be due to the compact tissue structure generating a higher fluid pressure, indirectly suggesting that most of the concentrations achieved in all the other extravascular tissues in the present study are delivered from the systemic circulation.

The goal of local vancomycin administration is to provide high target-site concentrations without reaching toxic systemic levels. To achieve this, the systemic absorption must be low. The present study could not confirm such a theoretical advantage of tibial intraosseous vancomycin administration. The mean peak drug concentrations in subcutaneous tissue and plasma were 2–3-fold lower in the present study than in a similar porcine study with intravenous administration of 1000 mg vancomycin (Bue et al., 2015). This raises an interesting question of whether an equivalent intraosseous dose of 1000 mg vancomycin would mirror the tissue concentrations from the intravenous setup but with higher tibial cancellous bone concentrations.

Vancomycin pharmacokinetics after intraosseous vancomycin administration have previously been examined in three clinical (250 and 500 mg; injection concentrations of 1.25, 2.5, and 3.33 mg/mL) and three experimental studies on rats (approximately 12.5 mg; injection concentration of 250 mg/mL), horses (300 mg; injection concentration of 5 mg/mL), and micropigs (approximately 350 mg; injection concentration of 17.5 mg/mL), respectively (Young et al., 2014; Loc-Carrillo et al., 2016; Rubio-Martinez et al., 2006; Chastagner et al., 2001; Chin et al., 2018; Young et al., 2018). Across these clinical and experimental studies, the mean peak drug plasma concentrations are reported in the range of 1.8 to 11 µg/mL, which is considerably lower than found in the present study (Young et al., 2014; Chin et al., 2018; Loc-Carrillo et al., 2016; Rubio-Martinez et al., 2006; Chastagner et al., 2001). Three of the studies (two clinical and one experimental) applied a proximal tourniquet before intraosseous administration, resulting in systemic vancomycin plasma levels of 0.0–2.7 µg/mL during inflation and a 4–10-fold increase after deflation (range: 1.8–6.0 µg/mL) (Young et al., 2014; Chin et al., 2018; Rubio-Martinez et al., 2006). The differences in plasma levels between these studies and the present study may be explained by the use of a tourniquet, different vancomycin doses, interspecies differences, and different sampling methods. Bone concentrations of vancomycin were examined in four of the studies (three clinical and one experimental) by means of tissue specimens (Loc-Carrillo et al., 2016; Young et al., 2014; Chin et al., 2018; Young et al., 2018). These studies demonstrate parallel variations in vancomycin bone concentrations with the present study. Any further comparisons are, however, complicated for several reasons; e.g. no application of tourniquet or major surgery was performed in the present study, and different sampling techniques, sites, and species were investigated. This study is the first to perform a dynamic assessment of vancomycin concentrations in cancellous bone adjacent to the intraosseous cannula and simultaneously from multiple orthopaedically relevant compartments over 12 h.

This study has a number of limitations. First, the variation in cancellous bone concentrations may imply that the sample size was too small. However, the limited systemic and intra-pig variation may indicate otherwise. Second, although pigs resemble humans in terms of physiology, anatomy, and the composition of bone, the interspecies and bone age differences have an effect on the translation potential of the presented results (Swindle et al., 2012; Aerssens et al., 1998). The weight of the pigs was chosen in order to resemble average human adult body weight. However, a young female pig (aged 5 months) weighing approximately 80 kg is still juvenile. Third, although validated for each matrix, vancomycin dialysate and plasma concentrations were quantified on two different assays, which may limit generalizability to some extent. Finally, microdialysis remains a sampling technique and is therefore exposed to limitations associated with the calibration procedures and chemical assay (Scheller and Kolb, 1991).

In conclusion, tibial intraosseous administration of 500 mg diluted vancomycin, without the use of a tourniquet, was found to provide high mean concentrations, well above the MIC target of 4 µg/mL, suitable for antibiotic prophylaxis, in all subjects, in adjacent proximal tibial cancellous bone throughout a 12 h sampling period. However, the concentrations in cancellous bone exhibited a large and unpredictable inter-pig variation. The systemic absorption was prompt and high, mirroring an intravenous administration, while none of the remaining extravascular compartments were able to reach a concentration of 4 µg/mL in all subjects.

## Data Availability

The data that support the findings of this study are available from the corresponding author upon reasonable request.
